# Novel Insights into Systemic Hyaluronic Acid Therapy in Dogs with Osteoarthritis from an Exploratory Postmarketing Study: Clinical Improvements Linked to Biomarker Changes

**DOI:** 10.3390/ani15213140

**Published:** 2025-10-29

**Authors:** Jana Matonohová, Matěj Šimek, Vratislav Berka, Lucie Bystroňová, Iva Lžičařová, Daniela Rubanová, Lukáš Kubala, Vladimír Velebný, Kristina Nešporová

**Affiliations:** 1 Contipro a.s., 56102 Dolni Dobrouc, Czech Republic; jana.matonohova@contipro.com (J.M.); matej.simek@contipro.com (M.Š.); vratislav.berka@contipro.com (V.B.); lucie.bystronova@contipro.com (L.B.); vladimir.velebny@contipro.com (V.V.); 2 Independent Researcher, 25219 Rudna, Czech Republic; iva.lzicarova@gmail.com; 3 Department of Biophysics of the Immune System, Institute of Biophysics, Academy of Science of the Czech Republic, 61200 Brno, Czech Republic; rubanova@ibp.cz (D.R.); kubalal@ibp.cz (L.K.); 4 Department of Experimental Biology, Faculty of Science, Masaryk University, 60200 Brno, Czech Republic; 5 International Clinical Research Center—Center of Biomolecular and Cellular Engineering, St. Anne’s University Hospital Brno, 65691 Brno, Czech Republic

**Keywords:** osteoarthritis, dog, hyaluronic acid, intravenous, Bonharen, biomarkers, inflammation, oxidative stress

## Abstract

This prospective, single-arm, exploratory postmarketing study preliminarily evaluated the clinical response and plasma biomarker changes in 18 client-owned dogs with naturally occurring osteoarthritis (OA) treated with sodium hyaluronate (Bonharen). Patients received intravenous injections of Bonharen Intravenous at a dose of 0.15 mL/kg (1.3–1.6 mg/kg hyaluronic acid once a week for consecutive five weeks). Clinical parameters (lameness, joint pain, mobility, swelling) were assessed weekly and two weeks after the final dose was given via standardized scoring. The plasma concentrations of selected inflammatory, cartilage-related, and oxidative stress biomarkers were measured before treatment and two weeks after the final dose. Clinical improvement in lameness and/or joint pain on palpation was observed in nearly half of the patients. No clinical deterioration was recorded at any time point. Physical activity increased in all patients with reduced baseline activity. Significant decreases in the plasma levels of prostaglandin E_2_, Δ17-6-keto prostaglandin F1α, malondialdehyde, and hyaluronan were detected, indicating reduced systemic inflammation and oxidative stress. In addition, an increase in plasma hydroxybutyrate and decrease in the collagen-breakdown marker prolyl-hydroxyproline were observed. No adverse effects were reported. These findings suggest that intravenous hyaluronic acid (Bonharen) may represent a safe and promising component to multimodal OA management in dogs and demonstrate the feasibility of integrating plasma biomarkers in canine OA studies.

## Simple Summary

Osteoarthritis is a common and progressive joint disease in dogs, leading to pain, reduced mobility, and decreased quality of life. One treatment option involves the intravenous administration of hyaluronic acid—a naturally occurring substance in the body that plays an important role in joint function. Although this approach is already used by some veterinarians, data on its effectiveness and systemic effects are still limited. This study evaluated the clinical and biological effects of intravenous administration of Bonharen Intravenous, an authorised veterinary medicinal product containing medium molecular weight hyaluronic acid, in client-owned dogs with naturally occurring osteoarthritis. Clinical signs such as lameness and joint pain were assessed, along with activity levels and blood biomarkers related to inflammation, oxidative stress, and tissue degradation. The results showed clear clinical improvement, accompanied by a reduction in systemic markers of inflammation, oxidative stress, and tissue degradation. No adverse effects were observed. These findings suggest that intravenous hyaluronic acid may offer a safe and effective option to help dogs with osteoarthritis lead more comfortable and active lives.

## 1. Introduction

Osteoarthritis (OA) is a highly prevalent and progressive joint disease in dogs. It involves the degradation of articular cartilage, changes in subchondral bone, and inflammation of the synovial membrane, ultimately resulting in joint pain, stiffness, lameness, and reduced mobility, which in turn impairs overall activity and quality of life. The prevalence of OA in the canine population is estimated, according to recent studies, to be as high as 38–40% [1,2].

The primary diagnostic tool for OA is a set of clinical parameters assessed by veterinary clinicians combined with imaging methods [3]. Interestingly, analyses of biomolecules in plasma that can serve as biomarkers for OA are not yet routinely used in veterinary clinical practice. However, they are increasingly studied as objective tools for monitoring treatment response, overcoming the limitations of subjective clinical assessment and imaging [4]. Based on dog and human clinical studies several molecules, such as interleukin-6 (IL-6), tumor necrosis factor-alpha (TNF-α), prostaglandin E_2_ (PGE_2_), matrix metalloproteinase-3 (MMP-3), matrix metalloproteinase-13 (MMP-13), hyaluronic acid (HA), and malondialdehyde (MDA), have been proposed as promising biomarkers of inflammation, tissue catabolism, and oxidative stress in OA [5,6,7,8,9]. However, it can be speculated that also other biomolecules in plasma can reflect OA pathological processes, such as degradation products of the joint extracellular matrix or metabolites reflecting changes in cellular energetic metabolism.

Current management of canine OA relies on a multimodal approach that combines pharmacological treatment primarily with nonsteroidal anti-inflammatory drugs (NSAIDs), corticosteroids and adjunctive analgesics, rehabilitation, weight control, and dietary support [3]. However, the classical therapeutic approaches have limitations, mostly related to side effects or limited potential. A typical example is the use of NSAIDs, which long-term use, while effective for pain relief, can lead to gastrointestinal, renal, or hepatic side effects [10]. Therefore, novel strategies have been introduced such as the application of biologics in veterinary medicine, which is attracting growing attention and being applied more frequently. Monoclonal antibodies neutralizing nerve growth factor were approved for OA treatment in dogs (bedinvetmab) and cats (frunevetmab) [11,12]. However, animal species-specific biology of diseases, limited number of animals with specific disease, and financial costs represent barriers to broader use of monoclonal antibodies in veterinary practice [13]. Recently, in addition to traditional intra-articular (IA) corticosteroids, there is increasing use of IA therapies that include application of biomolecules that naturally form key components of the joint extracellular matrix, such as HA or collagen, or of blood derivatives, such as platelet rich plasma, or stem cells [14,15,16]. Surgical intervention is considered in advanced stages.

Focusing on the therapeutic application of HA, IA injections of HA aim to restore synovial fluid viscosity and provide joint lubrication. However, the procedure is rather invasive, often requiring sedation and posing risks of joint infection or trauma. In contrast, intravenous (IV) administration of HA presents a less invasive alternative, offering systemic distribution to multiple joints simultaneously, and previous studies have demonstrated the potential efficacy of IV HA in reducing inflammation in musculoskeletal disorders [17,18]. Although not formally included in current treatment guidelines, IV HA is used in veterinary practice for joint-related conditions, particularly OA in horses and increasingly in dogs. For example, Bonharen Intravenous is an authorised veterinary medicinal product containing medium-molecular-weight HA (10 mg/mL) manufactured by Contipro a.s. (Dolní Dobrouč, Czech Republic), administered by intravenous injection and indicated for the treatment of joint disorders associated with non-infectious synovitis in horses and dogs. To date, evidence for IV HA in canine OA is limited and somewhat conflicting—an early uncontrolled report suggested potent therapeutic effects of IV HA on synovitis and traumatic arthritis in racing greyhounds. IV HA treatment reduced joint inflammation, improved joint function, and enabled successful return to racing [19]. Contrasting results were found by a small placebo-controlled trial involving dogs undergoing knee surgery for naturally occurring OA. No significant differences between HA and placebo were noted in synovial fluid properties or serum biomarkers [20].

Given the contradictory findings of previous studies with the limited number of parameters evaluated, additional research is needed to better establish the role of IV HA in canine OA and include additional parameters for an improved assessment of the clinical disease status. In particular, no published studies have evaluated the efficacy of IV HA using not only clinical scoring but also biomarker assessment in a defined canine OA population. To address this knowledge gap, the present exploratory study aims to preliminarily evaluate the therapeutic effects of repeated IV application of medium-molecular-weight HA (Bonharen Intravenous) in dogs with naturally occurring OA via combination of traditional clinical assessment with biomarkers analysis.

## 2. Materials and Methods

### 2.1. Study Design and Animals

**Figure 1:**
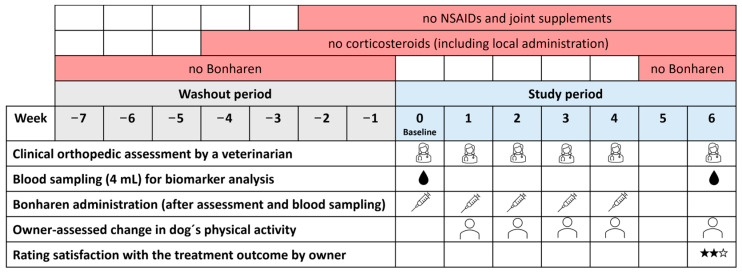
Experimental design and timeline of the study.

This was a prospective, open-label, single-arm, exploratory postmarketing study in client-owned dogs with naturally occurring OA. The study was aimed to evaluate clinical outcomes after treatment with Bonharen Intravenous which is an authorised veterinary medicinal product containing 300–600 kDa HA (10 mg/mL) manufactured by Contipro a.s. (Dolní Dobrouč, Czech Republic). The formulation of Bonharen Intravenous consists exclusively of sodium HA, sodium chloride, and water for injections. All dogs remained under the care of their owners during the study, and informed consent was obtained prior to study commencement. Ethical approval was not required for this study under Czech law (Act No. 246/1992 Sb.), as client-owned dogs were treated with a registered veterinary medicinal product (Bonharen Intravenous, Contipro a.s., Dolní Dobrouč, Czech Republic) following approved summary of product characteristics. Recruitment of patients, clinical evaluations, and administration of IV HA were performed by the private veterinary practitioner Dr. Iva Lžičařová under the guidance of the study protocol. Patients were eligible for the study if they met the following criteria: non-infectious synovitis, non-autoimmune OA, with clinical signs persisting ≥ 14 days and a lameness grade of 2–4 based on the grading scale described below; age 18 months to 12 years; and normal body condition score (BCS 2–3 on scale 1–5 [21]). In dogs with multiple affected joints, the most severely affected joint meeting the inclusion criteria was selected as the target joint for evaluation. The exclusion criteria included pregnancy, lactation, autoimmune synovitis, other joint diseases, HA metabolism disorders (such as cutaneous mucinosis in Shar-Peis), any other inflammatory conditions, or concurrent systemic illnesses. Other joint diseases, infectious or autoimmune synovitis, and systemic illnesses were ruled out by the attending veterinarian based on clinical examination, medical history, and, when indicated, standard diagnostic procedures such as imaging or laboratory tests. Prior to enrolment, patients required washout periods of ≥2 weeks for NSAIDs and joint supplements, ≥4 weeks for corticosteroids (including local administration), and ≥7 weeks for IV HA (Figure 1). Throughout the study, no medications, including NSAIDs, corticosteroids, or joint supplements, were permitted. Patients that required such treatments were withdrawn from the study to ensure appropriate care.

### 2.2. Study Protocol

At baseline (Week 0), the veterinarian performed a clinical evaluation, including lameness scoring, joint pain on palpation, joint mobility, and joint swelling assessment, and 4 mL of venous blood was collected for biomarker analysis. The same veterinarian conducted weekly clinical assessments (weeks 1–4) before each IV HA application and a final evaluation with blood sampling (4 mL) two weeks after the last dose (week 6). Clinical evaluations used predefined and consistent scoring (Table 1), with lameness assessed via CSU small animal orthopedic lameness grading scale as presented by Goh (World Small Animal Veterinary Association Congress Proceedings, 2019) [22]; joint pain on palpation and joint range of motion were scored according to the scoring system previously applied by Kampa et al. (2023) [23]; and joint swelling was evaluated using a simple custom scoring developed specifically for this study on the basis of routine veterinary practice.

At each visit except the first, dog owners assessed changes in their dog’s physical activity, and at the final visit they additionally rated their satisfaction with the treatment outcome, both using a questionnaire with predefined response options (Table 2).

Throughout the study, the veterinarian and dog owners monitored for any potential adverse events or suspected side effects, and they were instructed to report them if they occurred. Dogs were observed by the attending veterinarian for 1 h following administration and subsequently monitored by their owners. Given that the product is an approved veterinary medicinal product with a well-established safety profile, monitoring focused on physical signs only (such as local reaction at the injection site, behavior, and gastrointestinal, cardiovascular, and respiratory signs).

### 2.3. Treatment Protocol

Bonharen was administered intravenously into the saphenous vein at a rate of approximately 5 mL/min. The dosage followed the Summary of Product Characteristics (30–50 mg of sodium hyaluronate, i.e., 3–5 mL of the product per animal, depending on the size of the animal). For the purpose of the study, the standardised calculation of the dose 0.15 mL/kg body weight (rounded to the nearest 0.5 mL) was used. Accordingly, dogs weighing ≤ 20 kg received ≤ 3 mL. If the calculated dose exceeded the recommended maximum, the highest permitted dose of 5 mL was administered. Thus, the final administered dose (rounded to one decimal place) of HA was 1.3–1.6 mg/kg body weight (median 1.4 mg/kg). Each dog received five doses at seven-day intervals.

### 2.4. Blood Drawing and Plasma Separation

A fixed volume of 4 mL of blood was aseptically collected from each dog by venipuncture into tubes containing lithium heparin as an anticoagulant to ensure standardized sample handling and sufficient plasma for all planned biomarker analyses; this volume is well within the limits considered safe for clinical sampling. The samples were centrifuged at 13,000 rpm for 10 min to separate the plasma, which was subsequently aliquoted and immediately stored at −80 °C until analysis. Each aliquot was thawed only once prior to analysis to minimize analytical bias.

### 2.5. Quantification of the Plasma Concentrations of IL-6, TNF-α, MMP-3, and MMP-13

The plasma concentrations of IL-6, TNF-α, MMP-3, and MMP-13 were determined via commercially available enzyme-linked immunosorbent assay kits: canine IL-6 (RayBiotech, Inc., Peachtree Corners, GA, USA), Canine TNF-α (RayBiotech, Inc., Peachtree Corners, GA, USA), Canine Total MMP-3 (NovateinBio, Woburn, MA, USA), and Canine MMP-13 (Cusabio Technology LLC, Houston, TX, USA), following the manufacturers’ instructions. The limits of detection were 0.1 ng/mL for IL-6, 2 pg/mL for TNF-α, 0.15 ng/mL for MMP-3, and less than 0.039 ng/mL for MMP-13.

### 2.6. Quantification of HA and Chondroitin Sulphate

HA and chondroitin sulphate (CS) were quantified according to a modified method of Šimek et al. (2019) [24]. Briefly, ^13^C-labelled HA (Contipro a.s.), as an internal standard together with actinase E (Merck, Darmstadt, Germany), was added to the plasma samples to degrade the proteins. HA and CS were then depolymerized with a lyase from *Streptococcus pneumoniae* (Contipro a.s.), and disaccharides of HA and CS were quantified via liquid chromatography-mass spectrometry (LC–MS).

### 2.7. Quantification of Plasma Oxylipins, Pro-Hyp, and Hydroxybutyrate

Oxylipins, prolyl-hydroxyproline (Pro-Hyp) and hydroxybutyrate in plasma were analysed from isopropanol extracts containing deuterated primary COX and LOX MaxSpec^®^ LC-MS Mixture (Cayman Chemical Company, Ann Arbor, MI, USA) as internal standards. Oxylipin analysis was adopted from Chen & Zhang (2019) [25] and Chocholoušková et al. (2019) [26]. The samples were analysed with a Thermo Fisher Scientific TSQ Altis Plus mass spectrometer in negative mode. ACQUITY HSS T3 (1.8 μm, 2.1 × 100 mm, 40 °C) and water and acetonitrile, both modified with 0.02% acetic acid were used for separation. Gradient (0.3 mL/min) was as follows: 0–0.5 min—30% B, 0.5–1 min 40% B, 1–2.5 min 40% B, 2.5–4.5 min 40–70% B, 4.5–6.5 min 70% B, 6.5–9 min 95% B, 9–12 min 95% B, 12–12.1 min 95–30% B, 12.1–14.1 min 30% B. Analysis of Pro-Hyp and hydroxybutyrate was performed with Vanquish Horizon UHPLC system coupled with an Exploris 240 (Thermo Fisher Scientific, Waltham, MA, USA) mass spectrometer according to Shi et al. (2019) [27]. Mass spectometry data (*m*/*z* 70–800) were collected in negative ion mode using a dynamic exclusion list and a targeted inclusion list updated in the AcquireX data acquisition mode.

### 2.8. Quantification of MDA

The quantification of MDA was performed according to the modified method of Domijan et al. (2015) [28]. Briefly, 50 μL of plasma was mixed with 400 μL of 0.1% H_3_PO_4_ and 100 μL of 0.6% thiobarbituric acid and heated for 30 min at 90 °C. Afterward, the samples were cooled on ice and extracted with 3675 μL of a mixture of chloroform–methanol (4:1). The upper layer was then sampled and filtered through a 0.2 μm polyvinylidene difluoride syringe filter. The analysis was performed on an ACQUITY H-Class PLUS System using a fluorescence detector and an Acquity UPLC BEH C18 analytical column (50 × 2.1 mm, 1.7 μm). The mobile phases consisted of 50 mM KH_2_PO_4_ (pH 6.8) and methanol. The flow rate was 0.4 mL/min. The injection volume was 15 μL. Gradient: 0–3 min. 5% B to 40% B, 3–3.5 min 40% to 5% B, 3.5–4 min 5% B and 0.1 min equilibration. Column temperature: 40 °C. Excitation wavelength (λ): 527 nm; emission wavelength (λ): 551 nm.

### 2.9. Statistical Analysis

All statistical analyses were performed using GraphPad Prism 10 (GraphPad Software, San Diego, CA, USA). The distribution of plasma biomarker data was assessed using the Shapiro–Wilk test for normality. For normally distributed data, a paired *t*-test was used. For non-normally distributed data, the paired Wilcoxon signed-rank test was used. Differences were considered statistically significant at *p* < 0.05 or *p* < 0.01, as indicated in the Results.

## 3. Results

**Figure 2:**
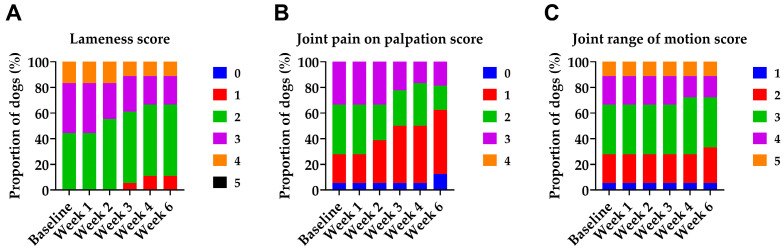
Distribution of clinical scores over time in dogs with OA during and two weeks after the last Bonharen dose (week 6). (**A**) Lameness score (0–5), (**B**) joint pain on palpation score (0–4), and (**C**) joint range of motion score (1–5). All the parameters were assessed by a veterinarian. The graphs show the proportion of patients assigned to each clinical score category at each visit. No patient exhibited any deterioration in lameness, pain sensitivity, or joint mobility at any time point during the study, even transiently.

Among the 25 dogs screened, 22 met the inclusion criteria and were included in the study. Three dogs were excluded during screening: two due to too mild lameness and one due to atopy, considered an inflammatory condition. Four dogs were excluded during the study: three due to injury (two of which required protocol-incompatible medication) and one due to clinical signs initially suggestive of sepsis; subsequent diagnostics revealed Steroid-Responsive Meningitis-Arteritis. Thus, 18 dogs (9 females, 9 males) completed the study. The median age was 10 years (range 2–12), and the median weight was 35 kg (range 15–40). The breeds represented were nine (50%) German Shepherd Dogs, four (22%) Belgian Malinois, two (11%) White Swiss Shepherd Dogs, one (5%) Weimaraner, one (5%) Chesapeake Bay Retriever, and one (5%) mixed-breed dog. In most dogs, osteoarthritis of the target joint was associated with developmental hip dysplasia, previous musculoskeletal trauma, excessive training, or age-related degeneration. The radiographic grade of OA was available for 16 of 18 dogs, with a median grade of 3 (range 1–4). The median duration of clinical OA signs was 3 years (range 1–8 years). The distribution of baseline clinical scores for lameness, joint pain on palpation, and joint range of motion is shown in Figure 2. Joint swelling was present in only one patient at baseline (classified as firm swelling without increased local temperature).

### 3.1. Clinical Assessments

In response to IV HA, we observed improvements in lameness, pain on palpation, and joint mobility (Figure 2). IV HA improved the lameness scores of 7 out of 18 patients (39%), with at least one case of improvement recorded at each baseline lameness grade included in the study (grades 2–4). All improvements were of a single grade and occurred between the second and fourth doses. In the case of joint pain on palpation, IV HA improved pain in 8 out of 17 patients (47%) with baseline pain across all recorded baseline grades (1–3). Most improvements were by one grade, with one case showing a two-grade improvement. These changes were typically observed after the second or third dose. Concerning joint mobility, significant improvement was observed in 2 out of 17 patients (12%), with a restricted range of motion at baseline. Both showed one-grade improvement. Joint swelling was present in only one patient at baseline (classified as firm swelling without increased local temperature) and remained unchanged throughout the study. Notably, no patient exhibited deterioration in any clinical parameter at any point during the study, not even transiently. Accordingly, no new swelling developed during treatment.

**Figure 3:**
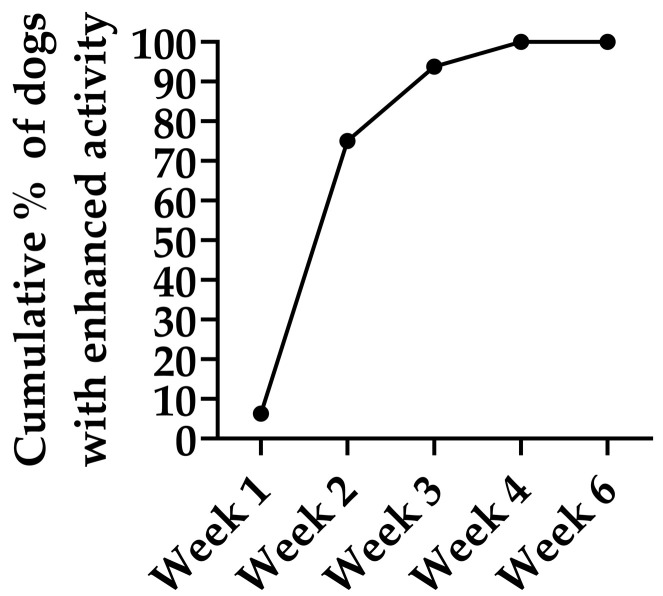
Cumulative percentage of dogs showing improved physical activity during and two weeks after (week 6) the IV HA treatment. Data represent owner-reported improvements compared with baseline in dogs that exhibited reduced physical activity at study entry (*n* = 16). Progressive improvement was observed, with 75% of the patients showing enhanced activity by Week 2, 93% by Week 3 and 100% by Week 4. This maximum improvement was sustained throughout the posttreatment follow-up period (Week 6), demonstrating progressive and sustained improvement in owner-perceived physical activity following Bonharen treatment.

Owner-reported physical activity increased in all 16 dogs treated with IV HA, which presented reduced activity prior to treatment (Figure 3). Progressive improvement was observed, with 75% of the patients showing enhanced activity by Week 2, 93% by Week 3 and 100% by Week 4. Most improvements were reported after the second dose and persisted throughout the study. The maximum improvement was sustained through the posttreatment follow-up period (Week 6), demonstrating progressive and sustained improvement in owner-perceived physical activity following IV HA treatment. No owner reported a decline in activity at any time point.

At the final visit, two weeks after the last IV HA dose, 14 owners (78%) reported being very satisfied with the treatment, 3 (17%) were satisfied, and 1 (6%) reported no treatment effect. The latter case involved a patient with only mild clinical signs at baseline and little room for measurable improvement. Neither the attending veterinarian nor the dog owners reported any adverse events related to IV HA during study therapy.

### 3.2. Biomarkers in Plasma

**Figure 4:**
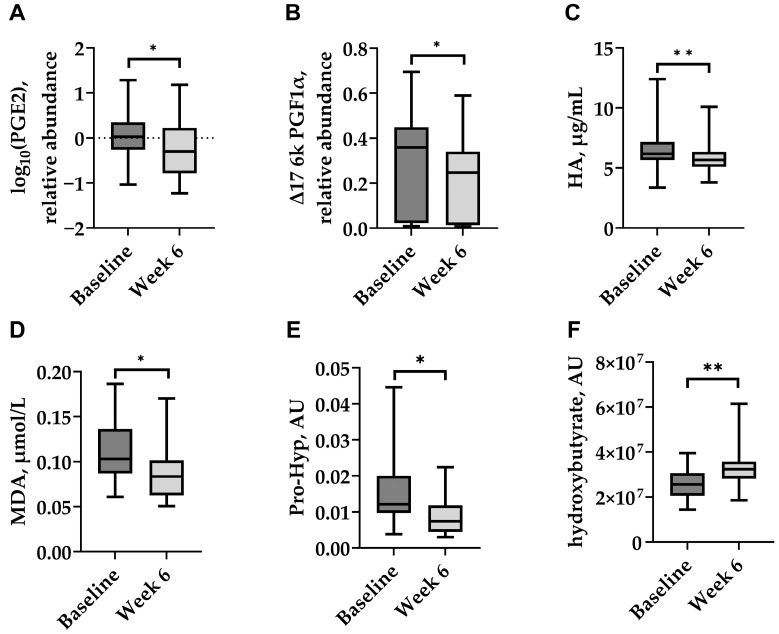
Plasma levels of selected biomarkers in dogs with naturally occurring OA at baseline and two weeks after the last Bonharen dose (week 6): (**A**) prostaglandin E_2_ (PGE_2_), (**B**) Δ17-6-keto prostaglandin F1α (Δ17 6k PGF1α), (**C**) hyaluronic acid (HA), (**D**) malondialdehyde (MDA), (**E**) prolyl-hydroxyproline (Pro-Hyp), and (**F**) hydroxybutyrate. Box plots show median (centre line), interquartile range (box), and minimum/maximum values (whiskers). Statistical analysis was performed using a paired Wilcoxon signed-rank test for (**A**,**C**,**E**,**F**), and a paired *t*-test for (**B**,**D**). * *p* < 0.05; ** *p* < 0.01.

The plasma concentrations of selected inflammatory and cartilage-related biomarkers, as well as a biomarker of oxidative stress, were measured before treatment and two weeks after the final dose (Week 6). IV HA administration resulted in significant reductions in PGE_2_ and Δ17 6k PGF1α (both *p* < 0.05; Figure 4A,B), as well as HA and MDA (*p* < 0.01 and *p* < 0.05, respectively; Figure 4C,D). Pro-Hyp, a marker of collagen degradation, also decreased significantly (*p* < 0.05; Figure 4E). In contrast, a significant increase in the plasma hydroxybutyrate level was detected following treatment (*p* < 0.05; Figure 4F). Other cartilage matrix markers showed no significant changes, with CS and MMP-13 concentrations remaining stable throughout the study.

The levels of IL-6, TNF-α, and MMP-3 were below the detection limits in most samples, despite the use of undiluted plasma. Specifically, IL-6 was undetectable in 15 dogs, and TNF-α and MMP-3 were below the quantification limits in 12 dogs.

## 4. Discussion

In this exploratory postmarketing study, we investigated the clinical efficacy of the veterinary formulation of medium-molecular-weight HA, Bonharen Intravenous, in client-owned dogs with naturally occurring OA. This represents the first comprehensive assessment in dogs that combines owner-reported outcomes, veterinary clinical evaluation and plasma biomarker analysis to evaluate systemic IV HA therapy under routine veterinary practice conditions.

IV HA was well tolerated in all animals, and no adverse reactions were reported by owners or observed by the attending veterinarian which aligns well with previous veterinary studies [18,29,30]. In terms of clinical outcome, improvements in lameness and/or joint pain on palpation were observed in nearly half of the treated patients with no deterioration in clinical signs at any point. Importantly, owners reported increased physical activity in all dogs with initially reduced activity levels and expressed a high level of satisfaction with the treatment outcome. The clinical improvements recorded in this study are consistent with previous veterinary observations of improved clinical symptoms of arthritis/synovitis in horses and dogs with musculoskeletal disorders mediated by IV HA treatment [19] and surgically induced OA [18,31]. On the other hand, a randomized, blinded, prospective placebo-controlled clinical trial in dogs with unilateral cranial cruciate ligament injury undergoing tibial plateau leveling osteotomy did not report significant effects of IV HA. Dogs with this natural joint pathology were sampled before osteotomy and at 2, 4 and 8 weeks postoperatively. They received three IV administration of HA, each dose containing 10 mg of HA (10 dogs) or 1 mL of 0.9% NaCl as placebo (12 dogs), administered immediately after osteotomy and at weeks 2 and 4. The lack of any significant change in synovial fluid viscosity, HA concentration, and serum glycosaminoglycan concentration may be caused by timing of blood collection, concurrent treatment of all dogs with NSAID Etodolac, and the administration of a uniform HA dose regardless of the dogs’ body weight [20]. The importance of appropriate dosing is also emphasized by pharmacokinetic studies, as high exogenous doses can saturate hepatic clearance, the primary pathway for HA elimination from circulation. This saturation may prolong HA’s half-life in the bloodstream, thereby enhancing its capacity to reach inflamed joints and exert the intended therapeutic effect [32,33,34].

In addition to clinical observations, significant reductions in prostaglandin E_2_ and ∆17-6-keto prostaglandin F1α were observed after systemic IV HA therapy. These biomarkers are products of cyclooxygenase pathways and reflect inflammatory processes in the organism at the systemic level. The decrease in inflammatory markers induced by IV HA is consistent with previous observations in different animal models [17,18]. Additionally, a significant decrease in plasma MDA, a marker of oxidative stress in OA [5], was detected in our study, consistent with previous reports of MDA level decrease in dogs with OA treated with a combination of meloxicam and tramadol [35], and in dogs with OA after supplementation with fish or corn oil [36], further supporting the protective anti-inflammatory effects of HA in OA.

In contrast, plasma MMP-13 and CS, although recognized as biomarkers associated with osteoarthritis [6,37,38], did not change significantly after IV HA treatment. This finding is consistent with previous reports in canine osteoarthritis models, where no significant alterations in serum MMP-13 [39] or in serum concentrations of the CS epitope CS846 [40] were observed following treatment. The lack of measurable changes in these markers may be attributed to the need for a longer treatment duration, more severe OA pathology, the use of synovial fluid rather than plasma for detection, or a combination of these factors. The levels of the proinflammatory cytokines IL-6 and TNF-α, as well as the proteolytic cytokine MMP-3, were below the detection limit in most plasma samples, preventing reliable interpretation.

The observed decline in systemic HA levels may initially appear paradoxical, given that HA was administered intravenously; however, it could reflect a reduction in joint inflammation and restored barrier integrity of the synovial membrane. Elevated HA concentrations in plasma have been associated with increased synovial permeability, cartilage damage, and clinical disease activity in human joint pathology [41,42,43,44]. Thus, the reduction in systemic HA may indicate normalization of joint homeostasis and reduced pathological HA turnover.

The plasma levels of Pro-Hyp, a dipeptide derived from collagen degradation, significantly decreased following Bonharen treatment. While Pro-Hyp is not a routine OA biomarker, previous studies have linked its presence in circulation to collagen turnover in conditions such as bone metastases and fibrotic tissue remodelling [45,46,47]. The observed decrease may suggest reduced matrix degradation, consistent with the therapeutic effect of HA.

Conversely, plasma hydroxybutyrate levels increased significantly. α-Hydroxybutyrate is a metabolite associated with fatty acid oxidation and has been identified as an exercise-induced marker in metabolomic studies [48,49]. Its elevation may reflect improved mobility or metabolic activation in response to reduced joint pain, aligning with owner-reported increases in physical activity.

While hydroxybutyrate and HA levels may be interpreted as evidence of increased activity and reduced inflammation, these metabolites are influenced by fasting status, stress, or broader metabolic conditions, which were not fully controlled in this exploratory study.

Overall, our findings suggest the potential value of integrating selected plasma biomarkers with clinical assessment in canine OA studies. While clinical signs provide essential real-world evidence, biomarker data can reveal underlying biological processes and treatment effects. This dual approach could enhance our understanding of disease dynamics and therapeutic response.

This exploratory study has several limitations due to its exploratory nature. The relatively small sample size and the absence of control and placebo groups constrain the strength of the conclusions. Due to the limited sample size, statistical correlation between radiographic OA grade and treatment response was not possible; however, descriptive data on OA severity were included to contextualize the clinical findings. Statistical analyses were limited to paired tests (*t*-test and Wilcoxon) without adjustment for multiple comparisons. This approach was considered appropriate given the exploratory nature of the study and the small sample size (*n* = 18), and the evaluation of only pre- and post- treatment samples for a restricted set of clinical and biomarker parameters. Future larger-scale studies with expanded sample sizes and more extensive parameter evaluation will be necessary to justify additional statistical adjustments and to validate these preliminary findings. Because all owners knew that their dogs received active treatment, a placebo effect cannot be ruled out. Nevertheless, the absence of deterioration and objective biomarker changes lend support to a genuine treatment effect. Biomarker analysis was performed using plasma rather than synovial fluid, potentially reducing sensitivity and joint specificity. While synovial fluid generally provides relatively high concentrations of joint-related markers, its invasive collection was deemed ethically inappropriate in privately owned dogs. Moreover, the sample volume further limits the range of biomarkers assessed. Future studies should include a broader panel of molecular markers to better characterize biological responses to treatment. As the laboratory analyses were not performed under randomized or blinded conditions, some degree of measurement bias cannot be excluded. Furthermore, the short follow-up period may have limited the detection of delayed or subtle effects, which is particularly relevant for chronic diseases such as OA. Further research is warranted to evaluate long-term outcomes. Additionally, clinical assessments were performed by a single unblinded veterinarian, which improved consistency but precluded interobserver variability analysis and may have introduced observer bias. However, the use of objective plasma biomarkers partially offsets this limitation. Furthermore, owner-reported activity and satisfaction questionnaires used are non-validated instruments. Standardized instruments such as the Canine Brief Pain Inventory (CBPI) or the Liverpool Osteoarthritis in Dogs (LOAD) were not used due to practical considerations and the need for a simple, easily completed questionnaire by owners. Lastly, although Bonharen Intravenous is a registered veterinary medicinal product, independent studies could further support these findings and strengthen the evidence for its use in clinical practice.

## 5. Conclusions

This study demonstrates that IV administration of medium-molecular-weight HA, Bonharen Intravenous, may represent a safe and promising option for dogs with naturally occurring OA. The treatment resulted in measurable clinical improvements, supported by owner-reported increased physical activity and favourable shifts in systemic biomarkers of inflammation, oxidative stress, and tissue degradation. The observed biomarker changes highlight the systemic biological activity of IV HA. These findings support the inclusion of systemic HA therapy within the multimodal management of canine OA.

## Figures and Tables

**Table 1:** Scoring system used for clinical orthopaedic assessment by a veterinarian.

Criterion	Clinical Scoring
Lameness	0 = no detectable lameness at any gait; 1 = barely perceptible lameness (discernible to a trained eye); 2 = mild or inconsistently apparent, weight-bearing lameness; 3 = moderate, obviously apparent, weight-bearing lameness; 4 = severe, predominantly weight-bearing lameness; 5 = severe, predominantly non-weight-bearing lameness.
Pain on palpation	0 = no pain; 1 = mild pain, dog turns head; 2 = moderate pain, dog retracts limb; 3 = severe pain, dog vocalizes or becomes aggressive; 4 = palpation not tolerated.
Joint range of motion	1 = full range of motion; 2 = slight restriction (10–20%), no crepitus; 3 = slight restriction (10–20%) with crepitus; 4 = moderate restriction (20–50%) with crepitus; 5 = severe restriction (>50%) with crepitus.
Joint swelling	1 = no swelling; 2 = soft swelling without increased local temperature; 3 = soft swelling with increased local temperature; 4 = firm swelling without increased local temperature; 5 = firm swelling with increased local temperature.

**Table 2:** Questionnaire items for owner-reported evaluation of treatment outcome and their satisfaction with it.

Criterion	Response Options
Change in dog’s physical activity	Increased activity No change Decreased activity
Satisfaction with treatment outcome	Very satisfied Satisfied No effect Condition worsened

## Data Availability

The original contributions presented in this study are included in the article. Further inquiries can be directed to the corresponding author.

## Abbreviations

The following abbreviations are used in this manuscript:

OA	Osteoarthritis
IL-6	Interleukin-6
TNF-α	Tumor necrosis factor-alpha
PGE_2_	Prostaglandin E_2_
MMP-3	Matrix metalloproteinase-3
HA	Hyaluronic acid
MDA	Malondialdehyde
NSAIDs	Nonsteroidal anti-inflammatory drugs
IA	Intra-articular
IV	Intravenous
MMP-13	Matrix metalloproteinase-13
CS	Chondroitin sulphate
LC-MS	Liquid chromatography-mass spectrometry
Pro-Hyp	Prolyl-hydroxyproline
Δ17 6k PGF1α	Δ17-6-keto prostaglandin F1α
MSCs	mesenchymal stem cells
